# College Note

**Published:** 1904-04

**Authors:** 


### COLLEGE NOTE.

The University of Buffalo will hold its fifty-eighth annual commencement early in May. A large and intelligent class will be graduated from the medical department. The other departments, too, are making correspondingly excellent showing for commencement day.

